# Screening of PI3K-Akt-targeting Drugs for Silkworm against *Bombyx mori* Nucleopolyhedrovirus

**DOI:** 10.3390/molecules24071260

**Published:** 2019-04-01

**Authors:** Bingbing Wang, Liang Jiang, Huizhen Guo, Qiang Sun, Yumei Wang, Enyu Xie, Qingyou Xia

**Affiliations:** 1 Biological Science Research Center, Southwest University, Chongqing 400716, China; wb7532@email.swu.edu.cn (B.W.); guohuizhen.111@gmail.com (H.G.); sq35@email.swu.edu.cn (Q.S.); wdwym7@email.swu.edu.cn (Y.W.); xieenyu@outlook.com (E.X.); 2 Chongqing Key Laboratory of Sericultural Science, Chongqing 400716, China; 3 Chongqing Engineering and Technology Research Center for Novel Silk Materials, Chongqing 400716, China

**Keywords:** antivirus, AZD8835, BmNPV, PI3K-Akt, silkworm

## Abstract

*Bombyx mori* nucleopolyhedrovirus (BmNPV) is the most prevalent threat to silkworms. Hence, there is a need for antiviral agents in sericulture. The PI3K-Akt pathway is essential for the efficient replication of the baculovirus. In an attempt to screen antiviral drugs against BmNPV, we summarized the commercial compounds targeting PI3K-Akt and selected the following seven oral drugs for further analyses: afuresertib, AZD8835, AMG319, HS173, AS605240, GDC0941, and BEZ235. Cell viability assay revealed that the cytotoxicity of these drugs at 10 µM concentration was not strong. Viral fluorescence observation and qPCR analysis showed that these candidate drugs significantly inhibited BmNPV in BmE cells. Only AMG319 and AZD8835 inhibited viral proliferation in silkworm larvae. The mortality of AZD8835-treated silkworms was lower than that of the control silkworms. Western blotting showed that AMG319 and AZD8835 decreased p-Akt expression after BmNPV infection. These results suggest that AZD8835 has application potential in sericulture.

## 1. Introduction

The silkworm is an important economic insect for silk production. Sericulture is one of the main sources of income for farmers in several developing countries [1,2]. However, silkworm diseases cause serious economic losses in sericulture. Viruses including *Bombyx mori* cytoplasmic polyhedrosis virus (BmCPV), *B. mori* densovirus (BmDNV), and *B. mori* nucleopolyhedrovirus (BmNPV), in particular, pose the most serious threat to silkworms [1]. Therefore, there is a need for antiviral agents in sericulture.

Breeding antiviral silkworm strains using transgenic technology is an approach employed for virus control [1]. For instance, overexpression of *Bmlipase-1* inhibits BmNPV during the initial infection stage [2]. Silencing of viral genes by RNAi suppresses the viral mRNA [3,4,5]. Overexpression of *hycu-ep32* inhibits viral protein synthesis [6]. Regulation of host immunity via overexpression of PGRPs enhances the antiviral capacity of silkworms [1,7]. Simultaneous overexpression of antiviral genes and knock-down of viral genes improves the resistance of transgenic silkworms [8]. Transgenic expression of Cas9 and gRNAs targeting the BmNPV genes also inhibits BmNPV in silkworms [9]. A safety assessment of transgenic silkworms has to be performed before commercialization. 

Antiviral drugs have been developed for coping with viral infection. Five compounds targeting viral proteins, namely, rimantadine, amantadine, zanamivir, oseltamivir, and baloxavir marboxil, have been approved by the FDA for inhibiting influenza virus [10,11]. Some host factors are involved in viral infection and are considered potential antiviral targets [10]. The PI3K-Akt signaling pathway plays important roles in cell survival, apoptosis, cell proliferation, and metabolic regulation, which can be activated by several viruses that modulate cellular events, and thereby augment viral replication [12]. For instance, influenza virus was able to induce Akt phosphorylation (p-Akt) in human lung carcinoma cells, and the viral multiplication was inhibited after treatment with the PI3K inhibitor LY294002 [13]. The PI3K-Akt pathway is also essential for the efficient replication of some viruses, such as human cytomegalovirus [14], coxsackievirus B3 [15], and baculovirus [12,16]. LY294002 inhibits *Autographa californica* multiple nucleopolyhedrovirus (AcMNPV) in Sf9 cells [12] and BmNPV in BmE cells [16]. AZD8835 is a novel dual PI3K inhibitor, which is currently in phase 1 clinical trials to treat breast cancer [17].

Gastrointestinal digestive juice is a chemical mixture of macromolecules, electrolytes, and enzymes, which interact to digest food and provide nutrients to animals [18]. Vertebrates strictly regulate the pH of the gastrointestinal tract by secreting hydrochloric acid in the stomach and bicarbonate in the pancreas, intestines, and cecum [19]. The digestive juice of lepidopteran insects is secreted by the midgut and affected by the K/H reverse transporter and the vacuolar-type proton pump in the goblet cells of the midgut epidermis, and the pH is as high as 11 [20,21]. The commercial oral inhibitors are designed and screened mainly based on the mammalian digestive system. In this study, we evaluated the inhibition ability of these PI3K-Akt-targeting drugs against BmNPV in BmE cells and silkworm larvae to analyze their application potential in sericulture. 

## 2. Materials and Methods

### 2.1. Silkworm Strain, Cells, and Viruses

Silkworm line Dazao (DZ), BmE cell line, wild BmNPV, and BmNPV-GFP expressing green fluorescent protein were maintained at the State Key Laboratory of Silkworm Genome Biology (Southwest University, Chongqing, China). BmE cells were cultured at 25 °C. Silkworms were fed with mulberry leaves under conditions of 25 °C and 12 h light/12 h dark. The silkworm larvae were orally inoculated with wild BmNPV and BmE cells were infected with BmNPV-GFP.

### 2.2. Inhibitors and Cell Toxicity Analysis

The PI3K inhibitors AZD8835, AMG319, HS173, and AS605240 were purchased from Selleck (Houston, TX, USA). The PI3K inhibitors GDC0941, LY294002, and BEZ235 and the Akt inhibitor afuresertib were purchased from MedChemExpress (Monmouth Junction, NJ, USA). Each drug was dissolved with dimethylsulfoxide (DMSO) to a storage solution of 100 mM. We diluted 100 mM of each drug with DMSO to a concentration of 10 mM of drug. BmE cells were seeded in 96-well plates 24 h before treatment with drugs. We added 10 mM of each drug to the cell culture medium at a dilution ratio of 1:1000 (0.1% of total volume) to prepare 10 µM of the drug. BmE cells were incubated with 10 µM of the drugs for 72 h. BmE cells were also incubated with 0 µM, 2.5 µM, 5 µM, 10 µM, 20 µM, 40 µM, 80 µM, and 100 µM of AZD8835 for 72 h. Each treatment with drugs and DMSO control comprised three replicates. Cell viability was measured using the CellTiter 96^®^ AQueous One Solution Cell Proliferation Assay (MTS) (Promega, Madison, WI, USA) [22].

### 2.3. Viral Infection and Drug Treatments 

BmE cells were infected with BmNPV at multiplicity of infection of 1 (MOI 1) and 10 µM drug. The infected cells treated with DMSO were used as the control. Viral fluorescence was observed at 72 h post infection (hpi). The third instar larvae were orally administered wild BmNPV (2 × 10^5^ occlusion bodies (OB)/larva) and drugs. The infected silkworms treated with DMSO were used as the infected control. There was also a non-infected control. Each treatment had three replicates and each replica consisted of 40 larvae. The mortality of larvae was recorded daily until day 10 post-infection [2,3]. Student′s *t*-tests were used to analyze the statistical data.

### 2.4. The qPCR and Western Blot Analysis

The total DNA was extracted at 48 hpi for the quantitative polymerase chain reaction (qPCR) analysis of the *GP41* gene of BmNPV and the control gene *GAPDH*. The average level of DMSO was set to 100%, and the values of drug treatments were normalized against this [2,3]. The total protein was extracted at 24 hpi, and p-Akt and the control GAPDH were detected by western blotting. 

## 3. Results

### 3.1. Summary of Drugs Targeting PI3K-Akt

Commercial compounds targeting PI3K-Akt are summarized in Table 1. Most of these are oral drugs, and only a small percentage are injectable. For use in sericulture, low-priced oral antiviral drugs with low IC50 are required. According to this principle, we selected seven candidate drugs from the commercial oral drugs, namely, the Akt inhibitor afuresertib and the PI3K inhibitors AZD8835, AMG319, HS173, AS605240, GDC0941, and BEZ235. Intravenous LY294002 was used as the control. The eight drugs were used in the subsequent analyses.

### 3.2. Analysis of Cytotoxicity of Candidate Drugs

**Figure 1:**
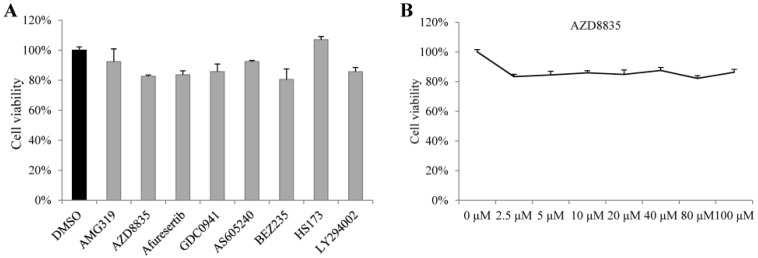
Cytotoxicity of candidate drugs. (**A**) BmE cells were incubated with 10 µM of each drug for 72 h. (**B**) BmE cells were incubated with different concentrations of AZD8835 for 72 h. The viability of cells was measured by the MTS assay. Bars, standard deviation.

In order to reduce costs, low doses of drugs are required to inhibit viruses in sericulture. Referring to the concentrations of these drugs used in other studies, 10 µM of each drug was chosen for detection in BmE cells. The viability of BmE cells treated with 10 µM AMG319, AZD8835, afuresertib, GDC0941, AS605240, BEZ235, HS173, and LY294002 was 92%, 83%, 84%, 86%, 92%, 80%, 107%, and 86%, respectively, compared with that of the cells treated with the DMSO control (Figure 1A), suggesting that the candidate drugs at 10 µM concentration do not have a strong cytotoxic effect. The cytotoxicity of AZD8835 in BmE cells was low upon treatment with 100 µM (Figure 1B).

### 3.3. Candidate Drugs Inhibited BmNPV in BmE Cells

**Figure 2:**
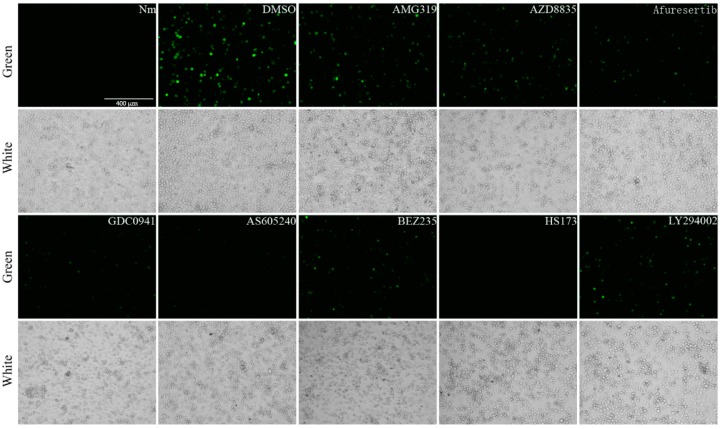
Observation of viral fluorescence at 72 hpi in BmE cells that were infected with BmNPV-GFP expressing green fluorescent protein at multiplicity of infection of 1 (MOI 1) and administered 10 µM of each drug. DMSO: the cells were infected with virus and dimethylsulfoxide (DMSO). Nm: non-infected control.

**Figure 3:**
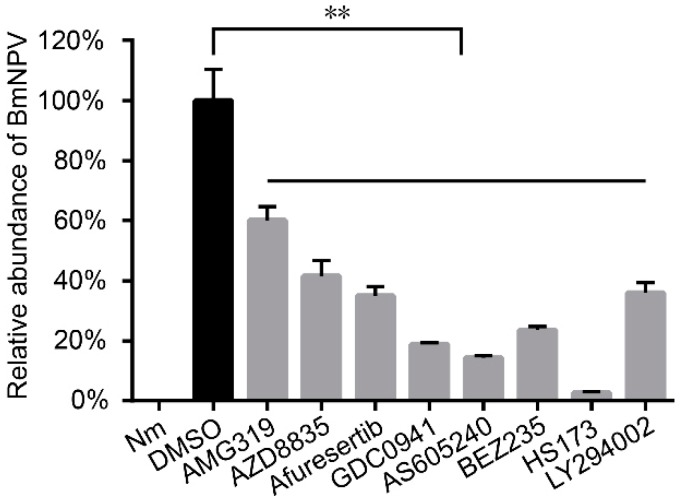
The quantitative polymerase chain reaction (qPCR) analysis of accumulated virus DNA at 48 hpi in BmE cells that were infected with BmNPV-GFP at MOI 1 and administered 10 µM of each drug. Bars: standard deviation; ** *p* < 0.01.

The virus fluorescence in drug-treated cells was obviously weaker than that in the control cells (Figure 2). The qPCR results showed that the drugs significantly inhibited BmNPV multiplication, and the accumulated virus DNA in AMG319-, AZD8835-, afuresertib-, GDC0941-, AS605240-, BEZ235-, HS173-, and LY294002-treated cells was 60%, 42%, 35%, 19%, 14%, 24%, 3%, and 36% compared with that of the DMSO-treated cells, respectively (Figure 3). These results revealed that the candidate drugs can inhibit BmNPV in BmE cells. 

### 3.4. AMG319 and AZD8835 Inhibited BmNPV in Silkworm

**Figure 4:**
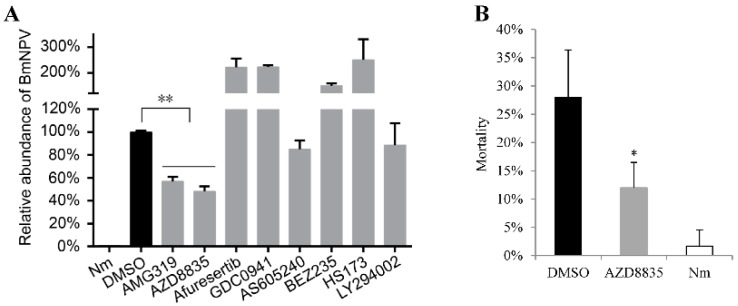
Analysis of the antiviral capacity of candidate drugs in treating silkworms. The third star larvae were orally infected with BmNPV and the drugs. DMSO: silkworms infected with virus and DMSO. Nm: non-infection control. (**A**) Analysis of the content of BmNPV DNA by qPCR. (**B**) Mortality. Bars: standard deviation; * *p* < 0.05 and ** *p* < 0.01.

The seven candidate drugs are administered orally either once or twice a day when used to treat humans or mice (Table 2). In this study, silkworms were treated daily with the same dose of each drug as is used to treat mice and humans. Silkworm larvae will die 4–5 days after infection with BmNPV. In order to reduce labor costs, silkworms were treated only once with high doses of drugs (for a total dose of 5 days). Therefore, the treatment doses of AMG319, AZD8835, afuresertib, GDC0941, AS605240, BEZ235, HS173, and LY294002 were 15, 125, 375, 375, 125, 125, 100, and 125 µg/g, respectively (Table 2). The third instar silkworm larvae were orally infected with BmNPV and administered a high dose of drugs only once. The qPCR results showed that afuresertib, GDC0941, AS605240, BEZ235, HS173, and LY294002 did not inhibit BmNPV, whereas AMG319 and AZD8835 significantly inhibited BmNPV in silkworms. The viral DNA content in AMG319- and AZD8835-treated silkworms was 57% and 48%, respectively, compared with that of DMSO-treated control cells (Figure 4A). AZD8835-treated silkworms were used for mortality analysis, and they showed significantly decreased mortality. The mortality of DMSO- and AZD8835-treated silkworms was 28% and 12%, respectively (Figure 4B). Almost all non-infected silkworms survived.

### 3.5. AMG319 and AZD8835 Decreased p-Akt after BmNPV Infection

**Figure 5:**
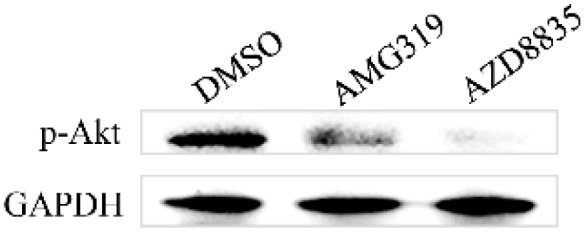
Detection of p-Akt by western blotting at 24 hpi in BmE cells that were infected with BmNPV-GFP at MOI 1 and administered 10 µM of each drug.

Western blotting showed that the p-Akt level was decreased in AMG319- and AZD8835-treated BmE cells when compared with that in the DMSO-treated control cells (Figure 5). A band of control GAPDH was detected, whereas the band of p-Akt was not observed in the total protein extracted from whole silkworms (data not shown). These results suggest that AMG319 and AZD8835 inhibit p-Akt after virus infection. 

## 4. Discussion

The PI3K-Akt pathway is required for the efficient replication of baculovirus [12,16]. BmNPV infects silkworm mostly via the oral route [1]. In this study, seven commercial oral drugs targeting PI3K-Akt were selected to determine their cytotoxicity and inhibitory effect on BmNPV. Among these, AZD8835 exhibited the strongest antiviral effect in BmE cells and silkworm larvae. 

Studies have reported that AcMNPV and BmNPV induced cellular p-Akt, and LY294002 inhibited induced p-Akt and viral proliferation in Sf9 and BmE cells [12,16]. The results of the present study show that LY294002 inhibits BmNPV in BmE cells (Figure 3) but not in silkworm larvae (Figure 4), as it is an intravenous drug. AMG319 and AZD8835 inhibited p-Akt in virus-infected BmE cells (Figure 5), but it was unexpected that p-Akt was not detected in whole silkworms. Further analysis revealed that p-Akt was detected in multiple tissues, but not in whole fifth instar larvae (data not shown). It is presumed that mulberry leaf consumption in whole silkworm affects the detection of p-Akt. Afuresertib, GDC0941, BEZ235, and HS173 significantly inhibited BmNPV in BmE cells (Figure 3). However, the viral content was increased after oral administration of these drugs in silkworms (Figure 4), which might be caused by drug toxicity to larval individuals. Differences in intestinal pH between insects and mammals might affect drug absorption and even cause drug toxicity. 

AZD8835 presented the strongest inhibitory effect on BmNPV among all the candidate drugs, and it reduced the mortality of silkworms to 16% after infection compared with that of the control (Figure 4). Whether AZD8835 can inhibit BmCPV and BmDNV in silkworms needs to be verified in the future. To develop drugs with higher antiviral capacity that could be applied in sericulture, the following aspects should be considered in future studies. First, optimize the structure of drugs to adapt to the digestion and absorption system of insects, which might increase absorption efficiency and decrease cytotoxicity [31]. Second, use carriers to promote the utilization of drugs. Polyamidoamine can be administered orally and stably in mice, which can improve the utilization of oral drugs [32]. Third, reduce the cost of drugs by controlling the purity of drugs and standards of the production process. Last, enhance the inhibitory effect of drugs on viruses by targeting multiple host factors, such as ERK, JNK [33], and PI3K-Akt, which are identified to be important for virus infection. 

In summary, we evaluated the inhibitory ability of seven oral drugs from the range of commercial PI3K-Akt-targeting drugs against BmNPV. AZD8835 inhibits viral proliferation in BmE cells and silkworm larvae after infection of BmNPV, indicating its potential application to decrease mortality in sericulture after further optimization. 

## Figures and Tables

**Table 1:** Summary of Drugs targeting PI3K-Akt.

Drug	Target	Stage	Style	Drug	Target	Stage	Style
Acalisib	PI3K	clinical	po. ^a^	HS173	PI3K	pre-clinical	po.
AMG319	PI3K	clinical	po.	NVPBGT226	PI3K	pre-clinical	po.
AZD8186	PI3K	clinical	po.	3CAI	Akt	pre-clinical	po.
AZD8835	PI3K	clinical	po.	AKT-IN-1	Akt	pre-clinical	po.
BEZ235	PI3K	clinical	po.	ARQ092	Akt	pre-clinical	po.
CH5132799	PI3K	clinical	po.	AT7867	Akt	pre-clinical	po./i.p.
Duvelisib	PI3K	clinical	po.	TIC10	Akt	pre-clinical	po./i.p.
GDC0941	PI3K	clinical	po.	A443654	Akt	pre-clinical	s.c. ^b^
GSK2636771	PI3K	clinical	po.	Deguelin	Akt	clinical	i.p. ^c^
Leniolisib	PI3K	clinical	po.	GSK690693	Akt	clinical	i.p.
LY3023414	PI3K	clinical	po.	SC66	Akt	clinical	i.p.
NVPBKM120	PI3K	clinical	po.	A66	PI3K	pre-clinical	i.p.
PF04691502	PI3K	clinical	po.	CNX1351	PI3K	pre-clinical	i.p.
PQR309	PI3K	clinical	po.	Isorhamnetin	PI3K	pre-clinical	i.p.
SAR245409	PI3K	clinical	po.	PI103	PI3K	pre-clinical	i.p.
VS5584	PI3K	clinical	po.	PIK75	PI3K	pre-clinical	i.p.
XL147	PI3K	clinical	po.	PIK90	PI3K	pre-clinical	i.p.
ZSTK474	PI3K	clinical	po.	SF2523	PI3K	pre-clinical	i.p.
afuresertib	Akt	clinical	po.	AKT inhibitor VIII	Akt	pre-clinical	i.p.
AT13148	Akt	clinical	po.	Akt1 and Akt2-IN-1	Akt	pre-clinical	i.p.
AZD5363	Akt	clinical	po.	BAY806946	PI3K	clinical	i.v. ^d^
GDC0068	Akt	clinical	po.	Gedatolisib	PI3K	clinical	i.v.
GSK2141795	Akt	clinical	po.	IPI549	PI3K	clinical	i.v.
MK2206	Akt	clinical	po.	TG100115	PI3K	clinical	i.v.
KRX0401	Akt	clinical	po.	LY294002	PI3K	pre-clinical	i.v.
AS605240	PI3K	pre-clinical	po.	PKI402	PI3K	pre-clinical	i.v.
CUDC907	PI3K	pre-clinical	po.	Wortmannin	PI3K	pre-clinical	i.v.
CZC24832	PI3K	pre-clinical	po.	3-Methyladenine	PI3K	pre-clinical	icv. ^e^
ETP46321	PI3K	pre-clinical	po.	IC87114	PI3K	pre-clinical	inhal ^f^

Note: ^a^ Peroral; ^b^ Subcutaneous; ^c^ Intraperitoneal; ^d^ Intravenous; ^e^ Intracerebroventricular; ^f^ Inhalational.

**Table 2:** Summary of candidate drugs.

Drug	References			Treated Silkworm		
	Species	Dosage (mg/kg)	Frequency	Stage	Dosage (µg/g)	Frequency
AMG319	rat	3	once/day	third instar	15	only once/total
AZD8835	mice	25	twice/day	third instar	125	only once/total
afuresertib	human	75	once/day	third instar	375	only once/total
GDC0941	mice	75	once/day	third instar	375	only once/total
AS605240	rat	25	once/day	third instar	125	only once/total
BEZ235	mice	25	twice/day	third instar	125	only once/total
HS173	mice	20	once/day	third instar	100	only once/total
LY294002	mouse	25	twice/day	third instar	125	only once/total

The data of AMG319 [23], AZD8835 [24], afuresertib [25], GDC0941 [26], AS605240 [27], BEZ235 [28], HS173 [29], and LY294002 [30] was from references.
